# Supplementary material for: Strength and Expansion of LHEC with Different Gypsum Contents Under Thermal Curing
Source: Materials (Basel). 2024 Nov 25;17(23):5766. doi: 10.3390/ma17235766 (PMC11642297; doi:10.3390/ma17235766)
Supplement: Supplementary file 1 [file materials-17-05766-s001.zip › materials-3251868-supplementary.pdf]

materials

Supplementary Materials

Strength and Expansion of LHEC with Different Gypsum Contents Under Thermal Curing

Bingxin Jin [1], Shuanglei Wu [1], Shujing Fan [2], Fafu Hang [2] and Huxing Chen [1,*]

[1] School of Materials Science and Engineering, Zhejiang University, Hangzhou 310027, China; 22226005@zju.edu.cn (B.J.); wushuanglei@zju.edu.cn (S.W.)
[2] Linhai Zhongxin New Building Materials Co., Ltd., Taizhou 317000, China; liuluorenjiantree@163.com (S.F.); hanghuohuo181030@163.com (F.H.)
* Correspondence: chenhx@zju.edu.cn; Tel.: +86-186-6817-9297

Citation: Jin, B.; Wu, S.; Fan, S.; Hang, F.; Chen, H. Strength and Expansion of LHEC with Different Gypsum Contents Under Thermal Curing. *Materials* **2024**, *17*, 5766. https://doi.org/10.3390/ma17235766

Academic Editor: João Pires

Received: 25 September 2024
Revised: 1 November 2024
Accepted: 15 November 2024
Published: 25 November 2024

Copyright: © 2024 by the authors. Licensee MDPI, Basel, Switzerland. This article is an open access article distributed under the terms and conditions of the Creative Commons Attribution (CC BY) license (https://creativecommons.org/licenses/by/4.0/).

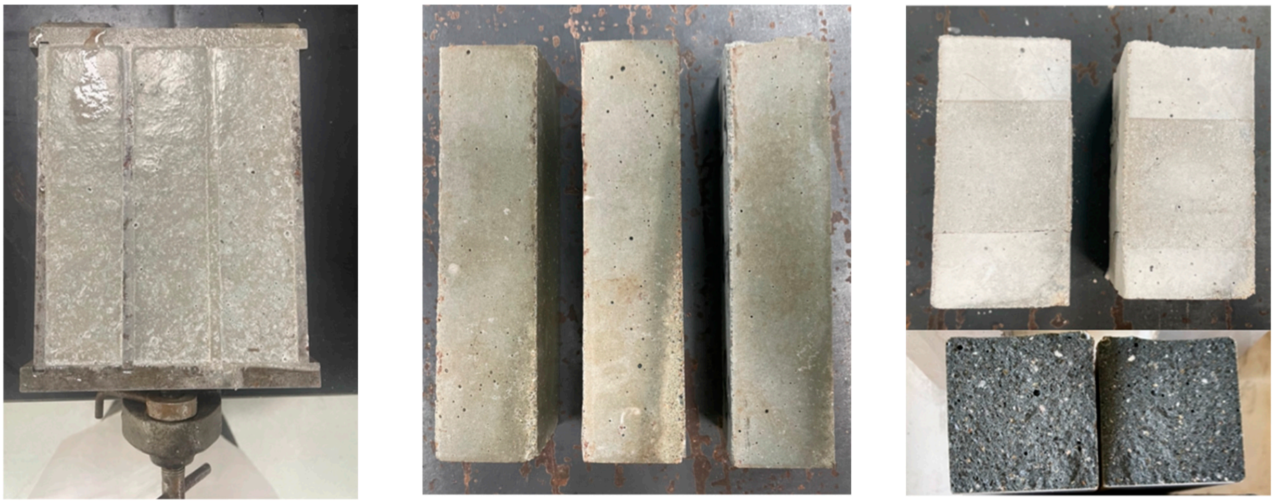

(**a**) (**b**) (**c**)

Figure S1. images of the experimental tests: (**a**) specimens before demolding; (**b**) specimens after demolding; (**c**) broken specimens after flexural strength test.
